# Molecularly Bridged Heterointerface Engineering: Designing Transparent, Wear‐Resistant, Hard yet Flexible Optoelectronic Protective Film

**DOI:** 10.1002/advs.74911

**Published:** 2026-03-19

**Authors:** Huiyang Lu, Zihui Liu, Jianan Yuan, Yunhao Wei, Shuangfei Xiang, Qinghua Lu

**Affiliations:** ^1^ State Key Laboratory of Synergistic Chem‐Bio Synthesis School of Chemistry and Chemical Engineering Shanghai Jiao Tong University Shanghai China; ^2^ School of Chemical Science and Engineering Tongji University Shanghai China; ^3^ School of Materials Science and Engineering Zhejiang Sci‐Tech University Hangzhou China; ^4^ State Key Laboratory of Micro‐Nano Engineering Science Shanghai Jiao Tong University Shanghai China

**Keywords:** bilayer‐structured film, coating, colorless polyimide (CPI), foldable cover window (FCW), polyhedral oligomeric silsesquioxane (POSS)

## Abstract

Transparent, hard yet flexible films serve as critical components for supporting and protecting flexible optoelectronic devices. However, current materials face significant technical challenges where polymer films exhibit low hardness and yield easily, and inorganic glass is brittle and inflexible. Herein, a bilayer‐structured transparent film with glass‐like hardness, polymer‐like flexibility, and ceramic‐like wear resistance is developed via a covalent bonding interface engineering strategy, employing a well‐defined polyhedral oligomeric silsesquioxane (POSS) coating as a hard layer, and a colorless polyimide substrate as a flexible layer. The PT10MMA coating, prepared by photo‐ or thermal‐crosslinking of its precursor T10‐MMA featuring a large inorganic cage, offers robust mechanical properties. The thermosensitive colorless polyimide (CPI) film supports the self‐crosslinking of T10‐MMA on it and interfacial crosslinking between the two, while also alleviating the stress‐induced fracture of PT10MMA and enhancing its strain tolerance. The robust heterogeneous interfacial bonding strength between PT10MMA and CPI layers ensures the retention of the inherent advantages of both layers, while also promoting the emergence of synergistic functionalities. Consequently, the film achieves >89.6% transmittance, 7H hardness, >500 steel wool abrasion cycles, creep‐free up to 50°C, and no creases after 200 000 bending cycles (1 mm bending radius).

## Graphical abstract

A molecular‐bridging heterogeneous interface engineering strategy is introduced to combine a polyhedral oligomeric silsesquioxane (POSS)‐based hard coating with a colorless polyimide (CPI)‐based flexible layer. The robust heterogeneous interface endows the integrated system with the superior properties of both layers and their synergistic functionalities, thereby effectively addressing the trade‐off among foldability, hardness, and wear resistance in transparent optical protective films.

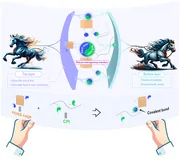

## 1 Introduction

Foldable displays overcome the spatial limitations of traditional flat displays, offering users an immersive visual experience and a more natural human‐machine interaction mode, thereby garnering widespread applications [1, 2, 3]. The foldable cover window (FCW), serving as the primary protective interface, plays a critical role in the implementation of bending, rolling, and folding of display devices, while ensuring clear and reliable imaging [4, 5, 6]. Therefore, FCW materials must simultaneously satisfy three fundamental requirements: (1) exceptional optical transparency, (2) sufficient flexibility to withstand cyclic tensile and compressive stresses during repeated folding operations, and (3) adequate surface hardness to protect the underlying display panel from mechanical damage, including impact, scratching, and fracture [7, 8, 9]. Unfortunately, these properties are typically mutually exclusive, particularly the inherent conflict between flexibility and hardness. Although inorganic glass and organic polymers have been employed as FCWs for foldable display devices [10, 11, 12], they each exhibit insurmountable drawbacks. Specifically, glass possesses extremely high hardness, yet it suffers from inherent brittleness and is prone to catastrophic failure under excessive mechanical stress [13, 14]. This hard yet brittle characteristic not only limits its standalone application in mobile phones that require extreme bending reliability but also makes it challenging to meet the demands of larger‐format devices that have higher requirements for lightweight and impact toughness, such as in emerging fields like foldable notebooks, rollable TVs, or automotive mobile displays. Conversely, polymer films demonstrate superior flexibility, yet their practical applications are limited by poor creep resistance and low hardness (<3H pencil hardness) [15]. These cases illustrate that achieving the coexistence of glass‐like hardness and polymer‐like flexibility in homogeneous networks is inherently challenging [16, 17], as the rigid glassy domains suppress polymer chain mobility, while the flexible segments compromise structural rigidity, a phenomenon known as the classical mechanical performance paradox [16, 18].

Extensive research efforts have been devoted to addressing the aforementioned challenges faced by FCW [7, 19, 20, 21]. The heterostructure lamination system, consisting of flexible polymer substrates and flexible hard coatings stacked together, has emerged as an effective strategy to fundamentally reconcile the conflict between hardness and flexibility [7, 22]. Compared to glass substrates, which are prone to fracture due to their rigid amorphous network structure and stress concentration at surface defects [5, 14], polymer‐based alternatives, such as polyethylene terephthalate (PET) [23], polycarbonate (PC) [24], polyethersulfone (PES) [25], polyamide (PA) [26], polyetheretherketone (PEEK) [27], polyethylene naphthalate (PEN) [28], and colorless polyimide (CPI) [29] exhibit superior operational reliability owing to their intrinsic flexibility through chain segment mobility and dynamic conformational changes. Among these, CPI films are the preferred choice for flexible substrates due to their exceptional mechanical robustness, optical transparency, and thermal stability [30]. As for coatings, organic materials such as epoxy resins [31], polyacrylates [32], and polyurethanes [33], as well as inorganic materials like silica nanoparticles [34] and silicon oxycarbide [35], typically provide either flexibility or hardness alone. Recently, coatings based on polyhedral oligomeric silsesquioxanes (POSS) with the molecular formula (RSiO_1.5_)*_n_* (where *n* = 8, 10, 12, etc.), featuring an organic–inorganic hybrid material, have attracted considerable attention due to their unique combination of hardness, wear resistance, and flexibility [20, 36, 37, 38]. “For example, Bae et al., utilizing a cycloaliphatic epoxy‐functionalized polysiloxane, pioneeringly demonstrated the potential of this emerging system as a coating for FCWs. Although this coating exhibits excellent performance, its preparation process is somewhat complex, requiring the close coordination of UV‐initiated ring‐opening polymerization and a subsequent high‐temperature, high‐humidity annealing process. Furthermore, during the process of achieving a high degree of curing, the volume of the coating undergoes shrinkage and re‐expansion, which inevitably introduces stress into the material, thereby posing a potential threat to the final adhesion strength and surface planarity of the coating to the substrate. More importantly, while the coating exhibits glass‐like hardness, it is also very brittle, and the mixed raw material system limits further improvement of this defect.” Despite significant breakthroughs having been achieved in CPI flexible substrates and POSS coatings, their heterogeneous integration remains challenging and has been rarely explored [20, 37]. This is primarily attributed to two critical technical obstacles: (1) insufficient understanding of the relationship between POSS cage size and its coating performance [4, 22, 39, 40, 41]. Previous studies revealed that cage dimension profoundly influenced their structural stability [41], photophysical properties [42, 43], dielectric characteristics [44, 45, 46], and gas permeability properties [47]. However, POSS coatings remain constrained by conventional siloxane mixture systems (containing T8‐T14 structures and their precursors; T represents RSiO_1.5_), which may inevitably suffer from structure‐property mismatches. This limitation creates significant barriers to achieving precise performance tuning and long‐term stability maintenance of such heterogeneous FCW films, and (2) interface failure of hetero‐integration induced by weak interfacial adhesion. This obstacle arises from the mismatch of surface energy, such as CPI using a fluorination strategy to enhance transparency [48], which inadvertently reduces the CPI's surface energy and increases its surface chemical inertness [49, 50]. This severely weakens the interfacial adhesion between POSS coatings and CPI substrates, causing severe failures such as warping, cracking, and even delamination of their heterogeneous interfaces under repeated folding cycles or harsh environmental conditions [51, 52]. Therefore, tailoring a POSS coating with a well‐defined cage size to achieve robust functionality and enhance its interfacial adhesion to CPI is a critical challenge that should be addressed to develop reliable FCW.

**FIGURE 1:**
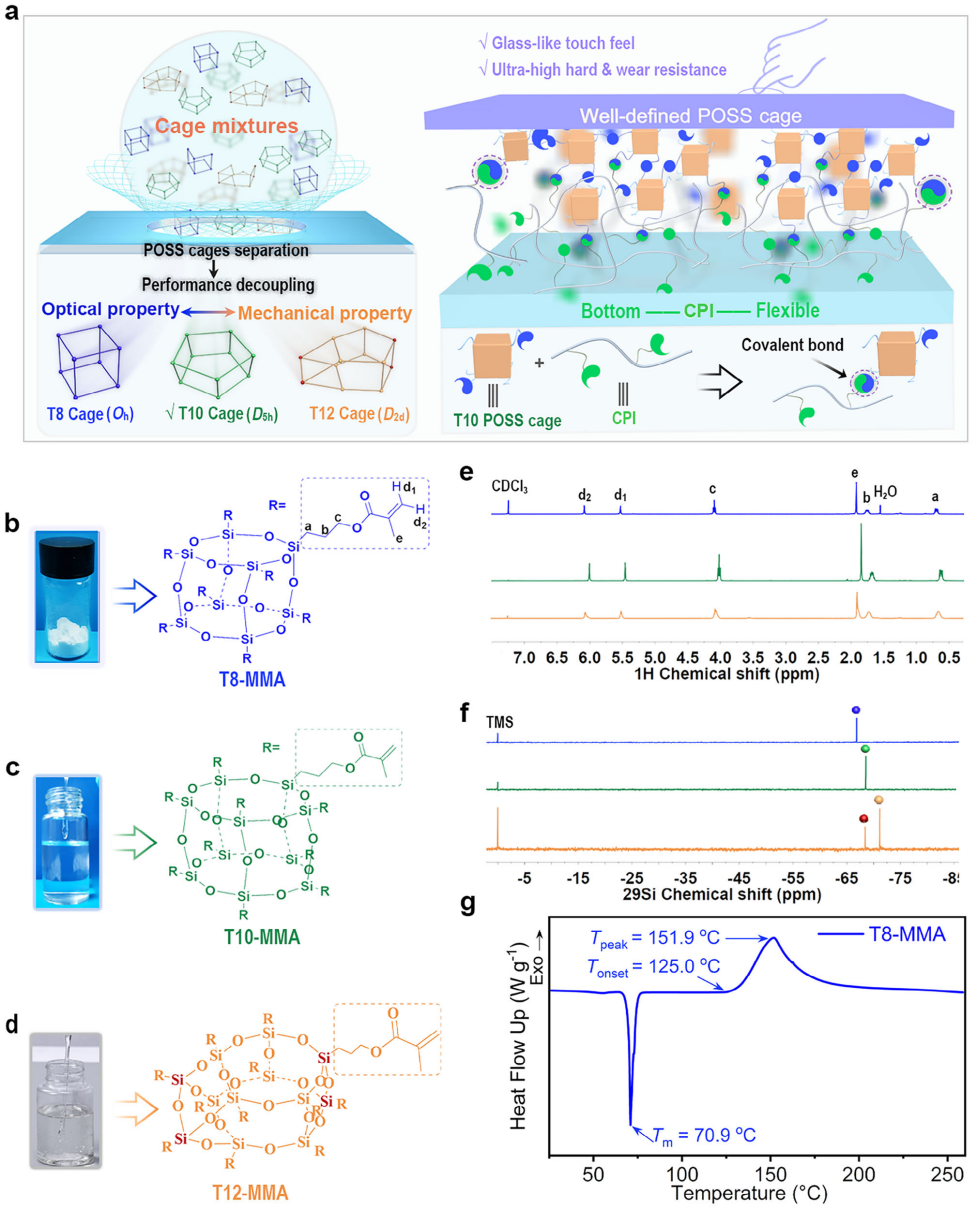
(a) Schematic illustration of POSS cage mixture separation, performance decoupling, and the design of a bilayer‐structured film, CPI‐PT10MMA. (b–d) The POSS monomers of (b) T8‐ MMA, (c) T10‐MMA, and (d) T12‐MMA. The red and yellow marks in the T12‐MMA molecular structure represent Si atoms with different ring tensions. (e) ^1^H NMR analysis. (f) ^29^Si NMR analysis. (g) DSC curve of T8‐ MMA.

Here, methacrylate‐functionalized octa‐, deca‐, and dodeca‐silsesquioxanes (T8‐MMA, T10‐MMA, and T12‐MMA) were prepared, and the differences in optical, surface, and mechanical properties of their cured POSS coatings (PT8MMA, PT10MMA, and PT12MMA) were systematically evaluated (Figure 1a, left). It was confirmed that only the PT10MMA coating achieved the optimal combination of performance properties, including optical transparency (>90% at 550 nm), pencil hardness (9H), wear resistance (1000 steel wool abrasion cycles), elastic recovery (*W*_e_ = 85%), and flexibility (*R* = 0.55 mm, coated on CPI film). Based on these findings, PT10MMA coating was covalently bonded to CPI film to construct a bilayer‐structured film, CPI‐PT10MMA (Figure 1a, right). The heterogeneous interfacial bonding strength of the CPI‐PT10MMA film is>71.0 MPa, far surpassing the adhesion strength of traditional polysiloxane coating technologies relying on van der Waals forces or hydrogen bonding. This robust interfacial engineering strategy reconciles the mutually exclusive properties of hardness and flexibility within a single material system, so the CPI‐PT10MMA film achieves a hardness of 7H, withstands >500 steel wool abrasion cycles, and maintains structural integrity under arbitrary bending and folding. Beyond these attributes, the synergistic effects arising from heterogeneous interfaces enable the CPI‐PT10MMA film to exhibit exceptional dimensional stability, as evidenced by its creep‐free behavior within the 40°C–50°C range and a strain recovery >90% at 70°C. Impressively, no cracking, delamination, or permanent yielding was detected in the CPI‐PT10MMA film even after undergoing 200 000 bending cycles. This material, by synergistically unifying a matrix of contradictory properties including optical transparency, high hardness, scratch resistance, excellent flexibility, and dimensional stability, exhibits unique application value unparalleled by UTG and CPI. It is poised to facilitate the transition of FCWs for large‐format devices from multilayer “sandwich” structures to ideal monolayer [53]. Furthermore, the high designability of the CPI‐PT10MMA material system promotes heterogeneous systems as a powerful platform to support next‐generation foldable optoelectronic technologies [54, 55].

## 2 Results and Discussion

### 2.1 Synthesis and Characterization of T8‐ MMA, T10‐ MMA and T12‐MMA

The methacrylate‐functionalized POSS cage mixture (T8‐MMA, T10‐MMA, and T12‐MMA) was synthesized via a nucleophilic substitution reaction of sodium methacrylate with octa(3‐chloropropyl)octasilsesquioxane, accompanied by a subsequent silsesquioxane cage rearrangement process (Scheme S1) [56]. And then each cage‐type product with a well‐defined structure was obtained through the methods of crystallization and silica gel column chromatography (Figure 1b–d). As shown in Figure 1e, the chemical shifts at ~5.54 and ~6.10 ppm are assigned to the double bonds of the methacrylate groups, which are observed in all POSS monomers. The ^13^C NMR spectra (Figure S1) indicate that the carbon atoms of T8‐MMA and T10‐MMA were in the same environment. Their specific structures were determined by ^29^Si NMR spectra (Figure 1f). The peaks at −66.837 and −68.612 ppm correspond to the Si atoms of the T8‐MMA (six symmetric 4‐membered rings, *O*_h_) and T10‐MMA (five 4‐membered rings and two 5‐membered rings, *D*_5h_) inorganic cages, respectively. Furthermore, the chemical shifts at −68.4410 and −71.1350 ppm for T12‐MMA indicate two different types of silicon atoms, corresponding to four 4‐membered rings and four 5‐membered rings (*D*_2d_), which are attributed to ring strain on the POSS cage [57]. Although POSS has large organic side chains, T8‐MMA is a crystalline solid, as demonstrated by XRD (Figure S2) at room temperature due to its high symmetry and close‐packed structure, while T10‐MMA and T12‐MMA are viscous liquids. The differential scanning calorimetry (DSC) curve of T8‐MMA revealed an endothermic peak at 70.9°C, attributed to its melting transition (Figure 1g). Notably, an intensive exothermic behavior was observed with an onset temperature ~125.0°C (peak maximum at 151.9°C), demonstrating that these photosensitive methacrylate groups are capable of thermal‐initiated polymerization.

### 2.2 Preparation of POSS Coatings

**FIGURE 2:**
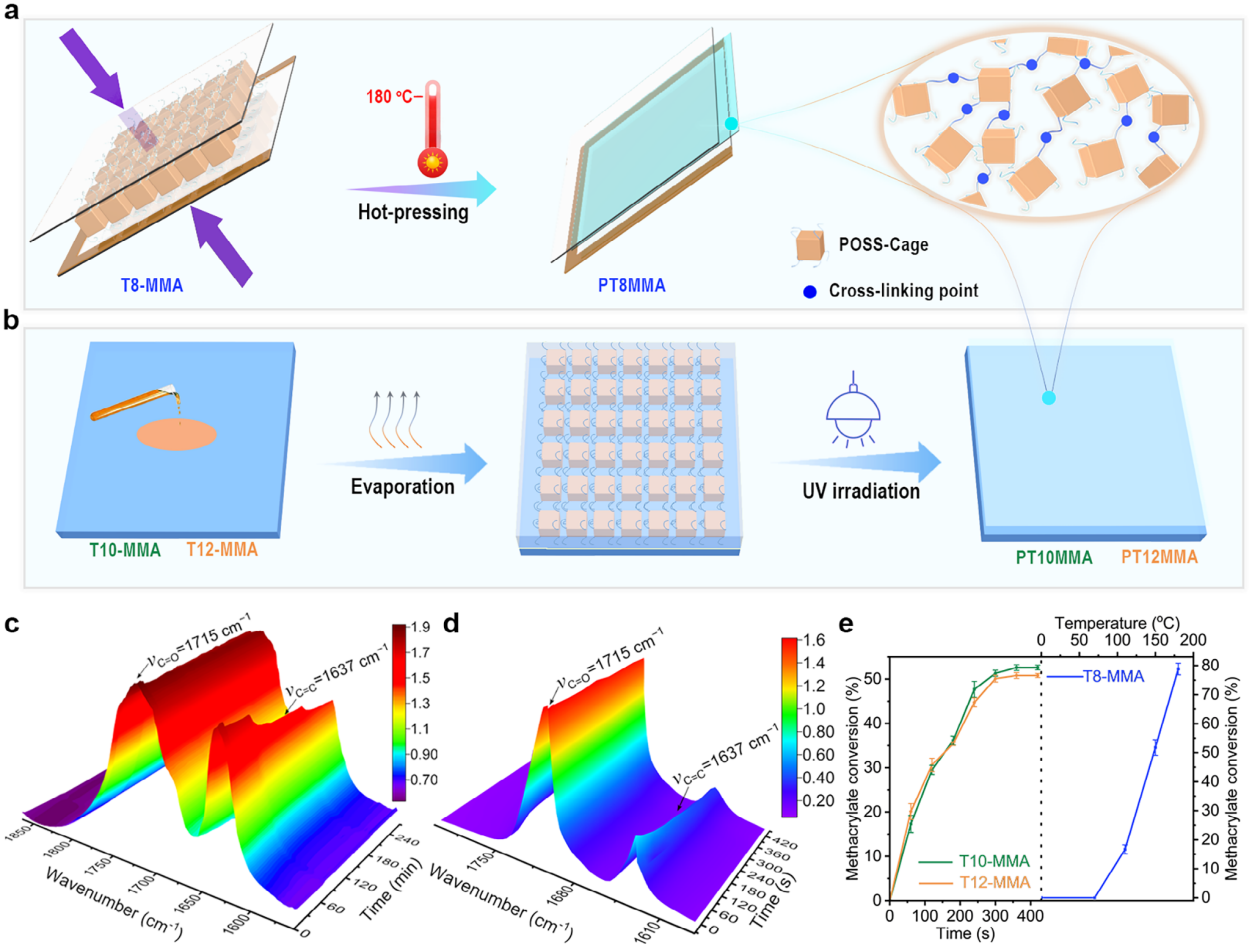
(a, b) POSS coatings prepared by (a) thermal curing for T8‐MMA, and (b) UV‐curing for T10‐MMA and T12‐MMA. (c, d) The time‐resolved FT–IR spectra of (c) thermal curing for T8‐MMA, and (d) UV‐curing for T10‐MMA. (e) Methacrylate conversion of T8‐MMA, T10‐MMA, and T12‐MMA as a function of curing time.

According to the physical state and photothermal characteristics of the designed POSS, thermal curing and photoinduced curing strategies were respectively employed to fabricate the coatings. For the highly crystalline T8‐MMA, transparent coatings (PT8MMA) were fabricated through a thermal compression process involving programmed temperature ramping to 180°C (Figure 2a). The coating thickness can be controlled by the applied pressure and the microcavity depth of the mold. Coatings from T10‐MMA and T12‐MMA can be prepared through thermal crosslinking curing or photo‐crosslinking curing (Figure 2b; Figure S3); however, considering processability and the necessity for achieving optimal surface uniformity, a photo‐curing method was used in this experiment. In brief, T10‐MMA or T12‐MMA and 1.0 wt.% photoinitiator DMPA were dissolved in PGEMA solvent, mixed evenly, and then coated on the substrate (glass or CPI) to prepare a coating by photo‐crosslinking after the solvent evaporates naturally. In this procedure, the coating thickness was modulated by varying the solution concentration.

The progress of UV‐curing of T10‐MMA and T12‐MMA, as well as the thermal curing of T8‐MMA, was monitored by using time‐resolved IR spectroscopy (Figure 2c,d). For the T10‐MMA and T12‐MMA monomers, the curing speed was obvious at the initial stage of UV‐irradiation due to the high freedom of methacrylate double bonds (1637 cm^−1^). As the curing process proceeds, the UV‐curing rate gradually decreases and eventually stabilizes and remains unchanged after 6 min. It is reasonable that the methacrylate double bonds cannot be completely converted, as the locally glassy domains and hard coatings formed by crosslinking hinder further reactions between double bonds [58]. The curing degree can be quantified by the methacrylate (C═C bond) conversion [59], with T10‐MMA and T12‐MMA reaching 51.3% and 50.1%, respectively (Figure 2e). On the other hand, the PT8MMA film was prepared by thermal crosslinking at 150°C, because the methacrylate conversion of T8‐MMA (51.7%) at this temperature is comparable to that of UV‐cured T10‐MMA and T12‐MMA. Although further elevating the temperature to 180°C can obtain a conversion of 78.8% (Figure 2e), excessive crosslinking degree results in stress‐mediated fracture of PT8MMA specimens (Figure S4). The wide‐angle X‐ray diffraction (WAXD) reveals the presence of two broad amorphous peaks in PT8MMA, which contrasts with the crystalline peaks observed in T8‐MMA, indicating that the ordered structure in T8‐MMA is disrupted during the thermal crosslinking polymerization process (Figure S5). Therefore, three decoupled hybrid materials (PT8MMA, PT10MMA, and PT12MMA) were successfully obtained by curing the corresponding POSS cage monomers, which provides the opportunity to systematically investigate their structure–property relationships.

### 2.3 Optical and Surface Properties of POSS Coatings

**FIGURE 3:**
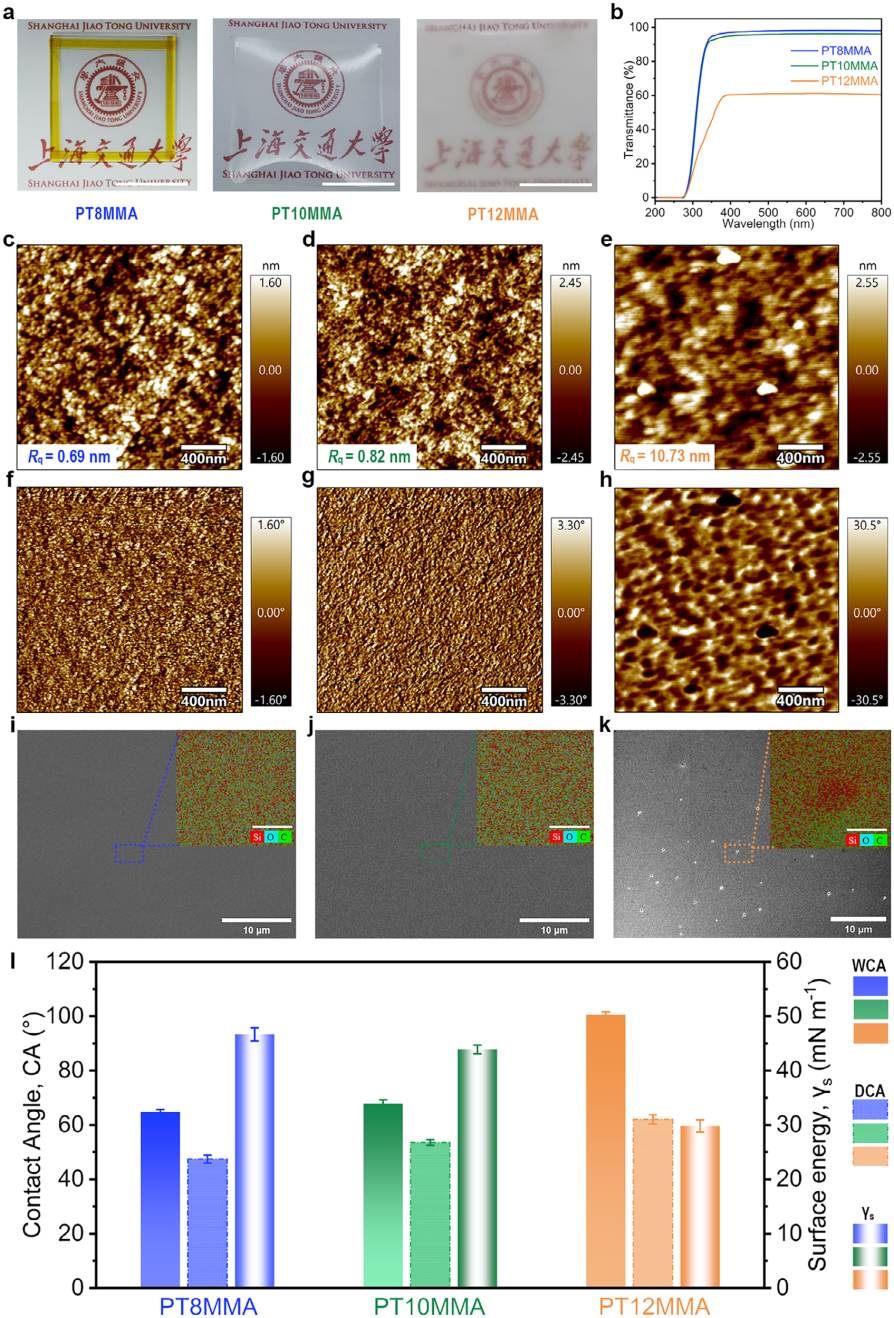
(a) Photographs of PT8MMA, PT10MMA, and PT12MMA coatings. Scale bar: 1.5 cm. (b) UV–vis spectra of three coatings (thickness: ~50 µm) on quartz glass substrates. (c–h) AFM images of (c, f) PT8MMA, (d, g) PT10MMA, and (e, h) PT12MMA coatings. (c–e) Height and (f–h) phase images. (i–k) SEM‐EDX images of (i) PT8MMA, (j) PT10MMA, and (k) PT12MMA. Scale bar for EDX images (insert): 500 nm. (l) WCA, DCA, and γ_s_ of PT8MMA, PT10MMA, and PT12MMA.

The surface morphology and structural characteristics of a coating are significantly correlated with its optical properties and surface wetting behavior. Therefore, these characteristics of the three types of coatings were systematically investigated. As shown in Figure 3a, both PT8MMA and PT10MMA coatings displayed exceptional optical clarity, enabling distinct visualization of the emblem through them, in stark contrast to the blurry PT12MMA coating. UV–vis spectroscopy revealed that the transmittance at a wavelength of 500 nm (*T*_500 nm_) is 98.6% for PT8MMA and 96.2% for PT10MMA coatings (quartz glass substrates), respectively (Figure 3b), indicating that they can provide high‐quality imaging for smartphones (Figure S6). However, the PT12MMA coating exhibited significantly inferior transparency, with a *T*_500 nm_ = 60.4%, far lower than that of PT8MMA and PT10MMA coatings. Since both the PT10MMA and PT12MMA coatings were prepared by photocuring, the differences in their optical properties do not originate from variations in the preparation process conditions but are more likely rooted in the fundamental dissimilarity in the POSS cage sizes of their constituent base monomers.

To investigate the underlying mechanisms responsible for the significant difference in optical performance, atomic force microscopy (AFM) was first employed to characterize the surface morphology of the three POSS coatings. AFM height images revealed ultra‐smooth surfaces for PT8MMA and PT10MMA coatings, with surface root mean square roughness (*R*_q_) values of 0.69 and 0.82 nm, respectively (Figure 3c,d), while the PT12MMA coating showed a markedly increased surface roughness (*R*_q_ = 10.73 nm) accompanied by pore‐like features (Figure 3e). Additionally, no phase separation was observed in both PT8MMA and PT10MMA coatings (Figure 3f,g), whereas the PT12MMA coating exhibited distinct phase contrast (Figure 3h). Scanning electron microscopy‐energy‐dispersive X‐ray spectroscopy (SEM‐EDX) analysis further confirmed that the surfaces of PT8MMA and PT10MMA coatings were highly smooth, with homogeneously distributed C, O, and Si elements (Figure 3i,j). However, the PT12MMA coating surface showed numerous non‐through pores structures (Figure 3k). It is these microstructural defects that enhance the scattering of incident light, thereby significantly reducing the optical transmittance of the PT12MMA coating. Notably, the morphological evolution and dimensional development of these pores are regulated by process parameters, specifically solvent properties and coating thickness (Figures S7 and S8 and Table S1). Although this phenomenon is intriguing, it falls beyond the focus of this study and therefore will not be extensively elaborated herein. The elemental distribution analysis of PT12MMA revealed a non‐uniform dispersion of C, O, and Si on its surface, with significant Si enrichment inside and around the pore structures (Figure 3k, insert). Due to the influence of the Si‐enriched porous structure, PT12MMA featured significantly different wetting properties compared to PT8MMA and PT10MMA (Figure 3l; Figure S9). The water contact angles (WCA) of PT8MMA and PT10MMA are 64.7° and 67.7°, respectively, indicating hydrophilic behavior. In contrast, the WCA of PT12MMA significantly increases to 100.5°, demonstrating hydrophobic characteristics. Furthermore, their oleophilic properties, as characterized by diiodomethane contact angles (DCA), follow the same trend: PT12MMA (62.0°) >PT10MMA (53.5°) >PT8MMA (47.4°). As a result, the surface energy (γ_s_) of PT12MMA is determined to be 29.8 mN m^−1^, which is markedly lower than that of PT8MMA (46.6 mN m^−1^) and PT10MMA (43.9 mN m^−1^). Although a low γ_s_ can effectively enhance the anti‐fouling performance of the PT12MMA coating, this characteristic inevitably compromises its effective wetting at the substrate interface and weakens the interfacial interactions between the two.

These results demonstrate that the inorganic cores (Si‐O‐Si) of smaller POSS cages (T8 and T10) possess excellent compatibility with their organic substituents, resulting in coatings with exceptional optical transparency. In contrast, coating based on a larger POSS cage (T12) shows phase separation and hydrophobic surface wetting behavior. These phenomena may be attributed to the differing structural stability and chemical properties of POSS with *O*_h_ (T8), *D*_5h_ symmetry (T10), and *D*_2d_ symmetry (T12) [41, 60]. This highlights the necessity of selecting POSS cage sizes for specific application scenarios in material engineering.

### 2.4 Mechanical Properties of POSS Coatings

**FIGURE 4:**
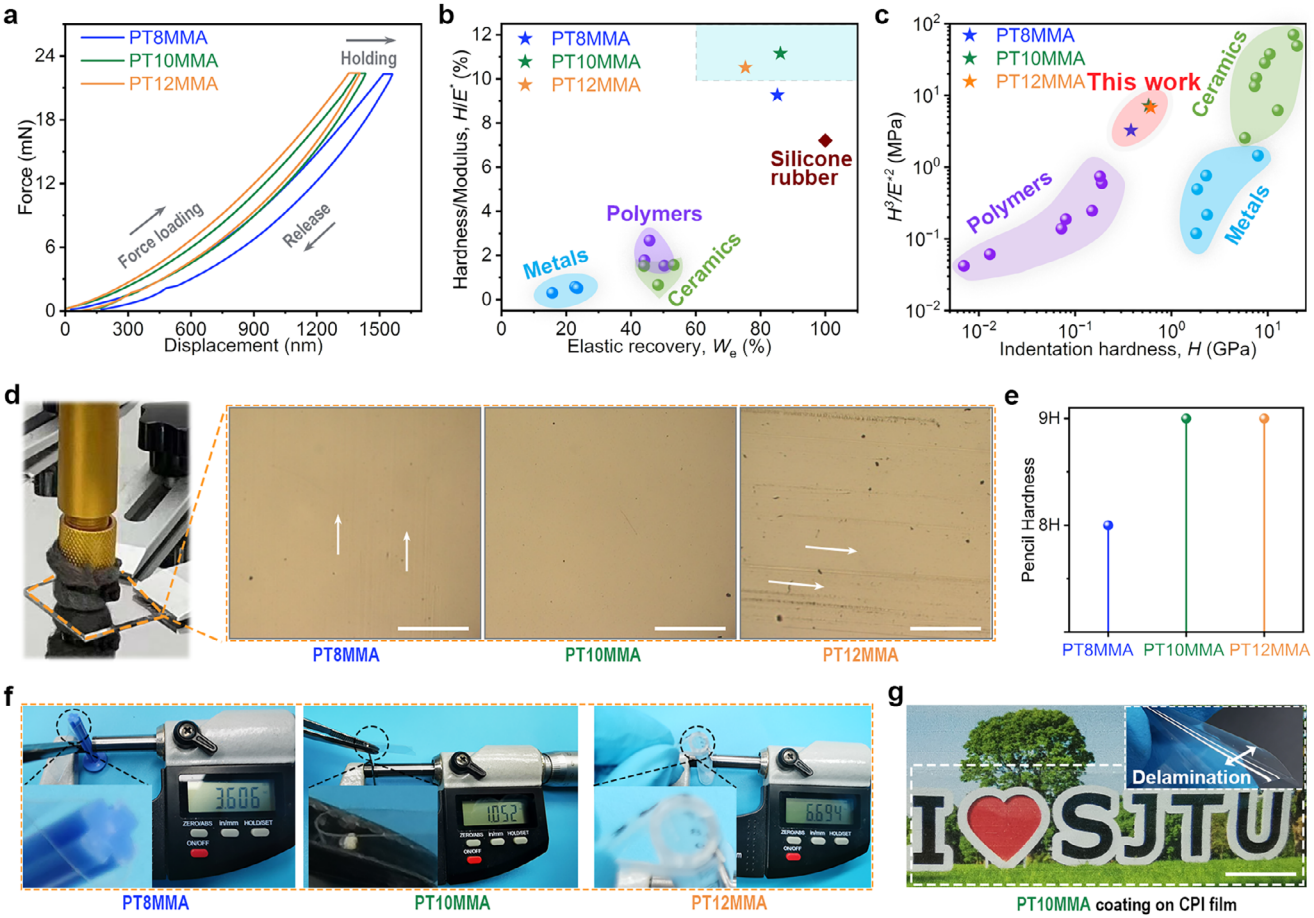
(a) Representative force *P*‐*h* curves of PT8MMA, PT10MMA, and PT12MMA coatings. (b, c) Comparison of (b) *H*/*E* versus *W*_e_ and (c) *H* ^3^/*E* ^2^ versus *H* for PT8MMA, PT10MMA, PT12MMA, and other typical engineering materials. (d) Photograph of the abrasion resistance tester and OM images of PT8MMA, PT10MMA, and PT12MMA coatings after 1000‐cycle steel wool abrasion under ∼10.0 kPa pressure. Scale bar, 500 µm. (e) Pencil hardness. (f) Photographs of PT8MMA, PT10MMA, and PT12MMA coating (~30 µm) on CPI film (~75 µm) in the bending test. (g) Photographs of PT10MMA coating on CPI film. Scale bar, 1.5 cm.

Robust mechanical properties, including strength, hardness, elastic recovery, and wear resistance, are essential to ensure durable device protection. Therefore, nanoindentation tests based on the Oliver‐Pharr method were performed to investigate these properties of the PT8MMA, PT10MMA, and PT12MMA coatings [61]. The force (*P*)‐displacement (*h*) curves show three stages of the force loading, holding, and release of the three coatings, respectively (Figure 4a). Under identical applied loads, a shallower indentation depth of the indenter into the coating indicates higher hardness. After load release, a smaller hysteresis (or the area enclosed by the *P*–*h* curve) in the *P*–*h* curve of the coating signifies better elastic recovery. As shown in Figure 4a, the PT12MMA coating exhibits the highest hardness, while the PT10MMA coating possesses the best elastic recovery. Quantitative analysis yielded indentation hardness (*H*), elastic recovery (*W*_e_), effective modulus (*E* ^*^), and wear resistance (*H* ^3^/*E* ^*2^) [62], which are listed in Table S2. As the size of the POSS cages increased, both the *E* ^*^ and *H* showed an increasing trend. This is attributed to the fact that the large‐sized POSS inorganic cores can impart enhanced mechanical properties to their hybrid coatings [44]. The *H*/*E* ^*^ and *W*_e_ values are commonly employed to evaluate the hardness and elastic resilience of coatings. When the *H*/*E* ^*^ and *W*_e_ values exceed 10% and 60%, respectively, the material is characterized as hard, flexible, and resilient [22]. Notably, conventional materials, including polymers, ceramics, metals, and rubbers, have a trade‐off between hardness and flexibility [17], as shown in Figure 4b. Among the three coatings, PT10MMA and PT12MMA exhibited *H*/*E* ^*^of 11.1% and 10.5%, with *W*_e_ of 86.1% and 75.3%, respectively. Both coatings met the above criteria, with PT10MMA demonstrating the best performance. Moreover, the scratch resistance of the coatings was assessed using the *H* ^3^/*E* ^*2^ value [17, 20]. The *H* ^3^/*E* ^*2^ values of the three coatings all surpass those of traditional engineering plastics and metals, and are even comparable to some ceramics, indicating a ceramic‐like scratch resistance (Figure 4c). Theoretically, PT10MMA and PT12MMA exhibit comparable wear resistance, both superior to PT8MMA. However, experimental results after 1000 cycles of steel wool abrasion showed an unexpected wear performance ranking: PT10MMA > PT8MMA > PT12MMA (Figure 4d; Figure S10). This discrepancy arises from the high surface roughness and the porous structure of PT12MMA, which increases frictional resistance and thereby accelerates its wear. Pencil hardness tests were conducted to evaluate their hardness, revealing that PT10MMA and PT12MMA achieve a hardness of 9H, while PT8MMA is slightly lower at 8H (Figure 4e; Figure S11). Despite their high hardness, these coatings should possess a modulus that is well‐matched with that of CPI, enabling the coatings to flexibly bend in tandem with the CPI substrate when applied without elastic mismatch (Tables S2 and S3) [20]. Subsequently, they were separately coated onto CPI film substrates to investigate their flexibility. The minimum bending radius (*R*) achievable without crack formation for PT8MMA, PT10MMA, and PT12MMA coated on CPI are 1.05, 0.51, and 3.26 mm, respectively, indicating that PT10MMA approaches near‐foldability (Figure 4f).

Through systematic investigations of the optical, surface, and mechanical properties of PT8MMA, PT10MMA, and PT12MMA, PT10MMA demonstrates the greatest potential as a hard, wear‐resistant, and flexible protective coating for FCW. Although this stacked system shows high transparency, unfortunately, spontaneous delamination between the PT10MMA coating and the CPI substrate occurred after three months under conditions of 25°C and ∼45% humidity (Figure 4g; Figure S12). This application obstacle caused by the weak interfacial adhesion highlights the necessity of improving heterogeneous interface engineering.

### 2.5 Fabrication of CPI‐PT10MMA Bilayer‐Structured Film

**FIGURE 5:**
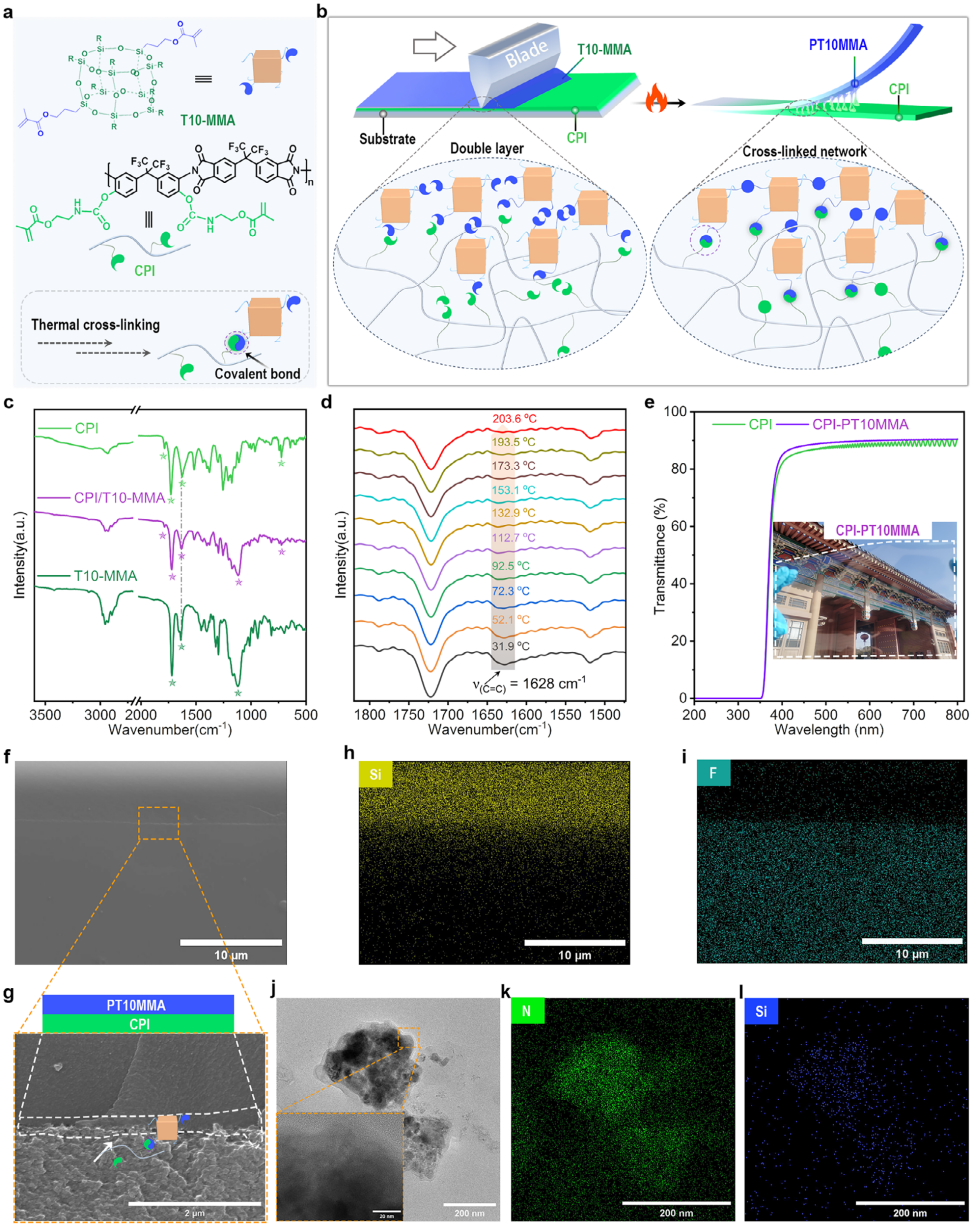
(a) Chemical structures of T10‐MMA and CPI, as well as the schematic illustration of the formation of covalent bonding via thermal activation. (b) Schematic illustration of a sequential blade‐coating process for fabricating bilayer structured composite film with programmed temperature elevation to facilitate solvent evaporation and crosslinking network formation. (c) FTIR spectra of T10MMA, CPI, and their blend CPI/T10MMA. (d) In situ FTIR spectra of cross‐linking of CPI and PT10MMA by thermal activation. (e) UV–vis spectra of CPI (75 µm) and CPI‐PT10MMA (115 µm) film. (f, g) SEM image showing the cross‐sectional view of the CPI‐PT10MMA film. (h, i) EDX elemental mappings of (h) Si and (i) F elements in (f). (j) TEM and HRTEM (insert) images of CPI‐PT10MMA film. (k, l) EDX elemental mappings of (k) N and (l) Si elements in (j).

To address the insufficient interfacial adhesion between POSS coatings and CPI substrate, a hard‐soft integrated bilayer‐structured film approach was proposed based on covalent bonding of the two components. The construction of this heterogeneous interface film involves grafting 2‐isocyanatoethyl methacrylate (ICEMA) onto CPI bearing phenolic hydroxyl side chains, enabling thermal crosslinking with T10‐MMA (Figure 5a), followed by the sequential blade‐coating of CPI and T10‐MMA solutions to form a wet double‐layer film and a final co‐heat treatment (Figure 5b). The synthesis of CPI is described in the Section 4, and detailed characterization is provided in the Supporting Information. The sequential blade‐coating process to fabricate the CPI‐PT10MMA film (Figure 5b and Section 4) is described as below: (1) a high‐solids‐content CPI resin solution was cast onto cleaned glass substrates and scraped to form a uniform wet film, (2) a low‐concentration T10‐MMA solution was subsequently coated onto the CPI layer, and (3) thermal treatment removed residual solvents and simultaneously initiated crosslinking of the heterogeneous interface. The thickness of each layer was controlled by adjusting solution concentrations and doctor blade heights during processing, while the CPI layer thickness was maintained at a fixed ~75 µm. Figure 5c shows the FTIR spectra of T10MMA, CPI, and their blend CPI/T10MMA. In the FTIR spectrum of CPI/T10MMA, the absorption peaks of different substances can be clearly distinguished: the Si─O─Si asymmetric stretching vibration (1116 cm^−1^) of T10‐MMA [20], the C═O asymmetric stretching vibration (1785 cm^−1^) and the C─N bending vibration (728 cm^−1^) of CPI [7], as well as the overlapping symmetric C═O stretching (1722 cm^−1^) and C═C stretching vibration (1628 cm^−1^) peaks of CPI and T10MMA. During the thermal cross‐linking process, the intensity change of the C═C group peak was used to evaluate the extent of cross‐linking [59]. In situ FTIR spectroscopy analysis revealed that the C═C group peak had essentially disappeared and remained unchanged thereafter upon heating the system to 193.5°C, indicating the completion of the crosslinking reaction (Figure 5d).

Remarkably, the optical transparency of the obtained CPI‐PT10MMA composite film (*T*_500 nm_ = 89.6%) was higher than that of the pristine CPI film (*T*_500 nm_ = 88.2%) (Figure 5e). This can be attributed to two reasons: (1) the reflectivity of PT10MMA is much lower than that of the CPI film (Figure S13), thereby enhancing the anti‐reflection performance of the CPI‐PT10MMA film, and (2) the use of pure T10‐MMA units mitigates the size effect of POSS cages, especially eliminating the crystallinity of the system (Figure S14). The cross‐sectional SEM image of the CPI‐PT10MMA film clearly shows the bilayer structure (Figure 5f,g). The cross‐sectional EDX analysis revealed mutual penetration between PT10MMA and CPI at the cross‐linked interface, forming a micron‐scale transition layer (Figure 5h,i), which is fundamentally distinct from the heterointerfaces observed in physically stacked architectures [63]. This transition layer helps to effectively transfer the internal and external stresses applied to the hard PT10MMA layer, thereby maintaining structural stability during deformation of the bilayer‐structured film [64, 65]. Transmission electron microscopy (TEM) was employed to evaluate the scale of interactions between hybrid siloxane and organic polymers in CPI‐PT10MMA. The TEM and high‐angle annular dark‐field scanning transmission electron microscopy (HAADF‐STEM) images of CPI‐PT10MMA show the presence of an amorphous hybrid network at the nanoscale (Figure 5j; Figure S15a). EDX mapping results indicate that a significant amount of N, O, and F elements are distributed around the Si element instead of agglomerating (Figure 5k,l; Figure S15b,c and Table S4), confirming the successful hybridization of the organic polymer and inorganic network at the heterointerface.

### 2.6 Structural Stability Assessment of CPI‐PT10MMA Film

**FIGURE 6:**
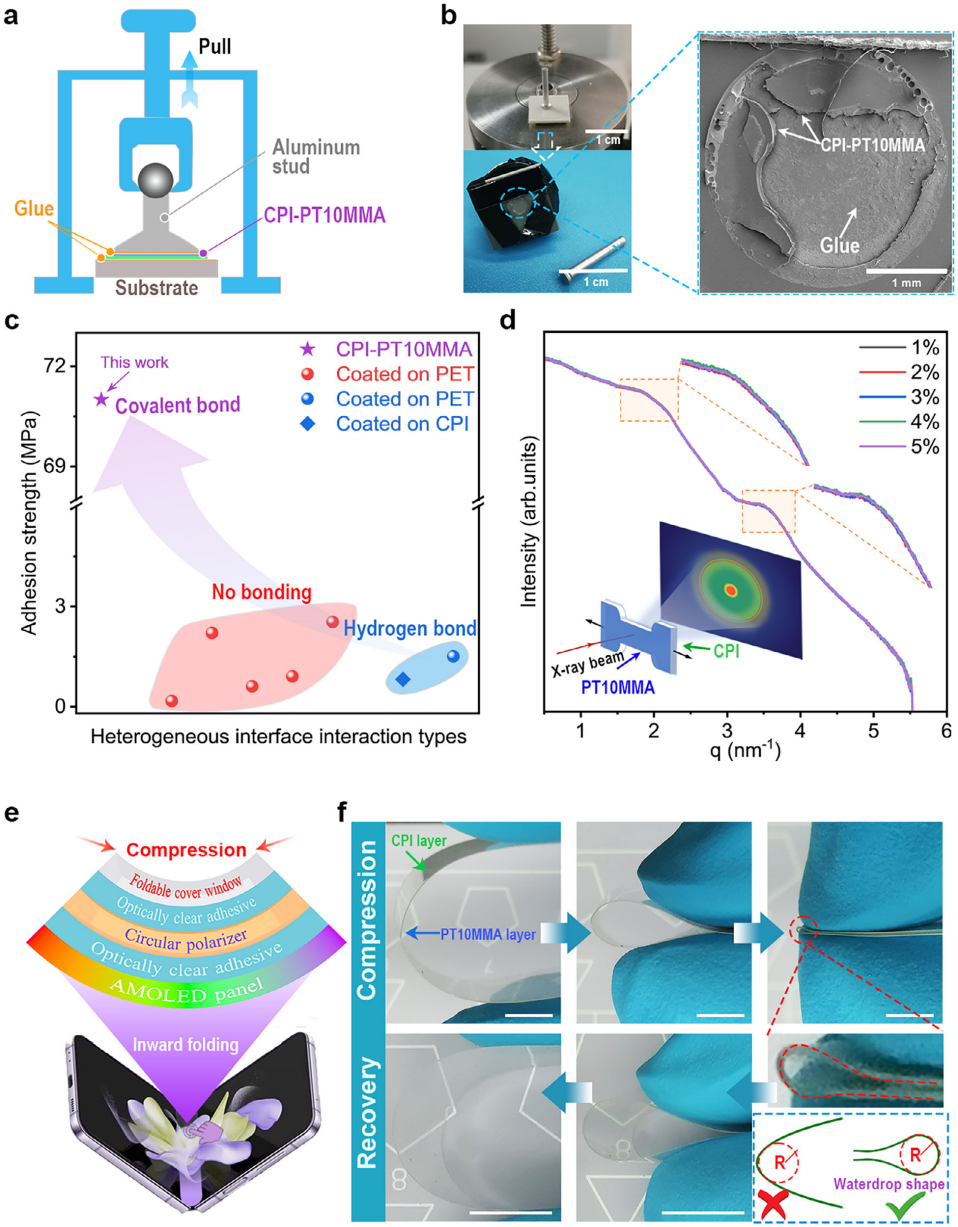
(a) Schematic illustration of the pull‐off test for CPI‐PT10MMA film. (b) Photographs of the CPI‐PT10MMA sample before and after the pull‐off test, along with the SEM image of its fracture surface. The thicknesses of the PT10MMA and CPI layers are ∼30 and ∼75 µm, respectively. (c) Comparison of heterogeneous interfacial adhesion strength between covalently bonded CPI‐PT10MMA films and representative non‐bonded or hydrogen‐bonded polysiloxane coatings on polymer substrates. (PET and CPI). (d) SAXS scattering intensity versus scattering vector (*q*) profiles of CPI‐PT10MMA film with different strains. (e) Schematic illustration of the impact of foldable device spatial transformation on display modules. (f) Photographs of inward bending and recovery of the CPI‐PT10MMA film.

Adhesion strength serves as a critical parameter for evaluating the separation resistance between coatings and substrates, and can reflect the durability of the composite film. As shown in Figure 6a, the pull‐off method was employed to test the debonding behavior and corresponding bonding strength between the PT10MMA layer with varying thicknesses (∼250, ∼150, ∼100, ∼50, and ∼30 µm) and a CPI (∼75 µm) layer (Figure S16). SEM observations of the pull‐off interfaces revealed distinct failure mechanisms dependent on the PT10MMA layer thickness. For specimens with a PT10MMA layer exceeding 50 µm, this layer would break, crack, and wrinkle under a small peeling force due to its brittleness, resulting in a low adhesion strength with the CPI layer (Figure 6b; Figure S17). However, when the PT10MMA layer thickness was reduced to 30 µm, no interfacial delamination between the CPI layer and the PT10MMA layer was observed at the pull‐off CPI‐PT10MMA film interface. In this case, the PT10MMA layer adhered firmly to the CPI layer and was pulled out together with the CPI layer, and their interfacial bonding strength was >71.0 MPa (Figure S18). Figure 6c and Figure S19 show a comparative analysis of heterogeneous interfacial bonding strengths for three characteristic adhesion mechanisms between the polysiloxane coatings and substrates (PET and CPI). The interlayer bonding strength of CPI‐PT10MMA film is dozens of times higher than that of non‐bonded (van der Waals) or hydrogen‐bonded polysiloxane coatings, demonstrating the superior advantages of covalent‐bonded heterointerface films. Small‐angle X‐ray scattering (SAXS) tensile testing revealed that as the strain of the CPI‐PT10MMA film increased from 1% to 5%, both the intensity and position of the scattering peaks at *q* = 2 nm^−1^ and *q* = 3.5 nm^−1^ remained unchanged (Figure 6d). This observation indicates that the CPI‐PT10MMA film can withstand large strains without undergoing structural degradation. When foldable devices alter their spatial configurations, the display modules are subjected to significant stresses, such as compressive stresses during inward folding (Figure 6e). Therefore, FCWs must possess elastic strain capabilities that adapt to the dynamic changes in folding devices to prevent mechanical failures such as cracking, delamination, or permanent deformation. As shown in Figure 6f, the CPI‐PT10MMA film gradually bends inward under compressive stress until it nearly achieves a complete fold, yet fully recovers its original shape upon stress release. This behavior is attributed to the high‐toughness PT10MMA layer, which suppresses arc‐shaped deformation during folding and instead promotes waterdrop‐shaped deformation. At the same *R*, the latter deformation mode ensures reversible shape recovery in the CPI‐PT10MMA film, effectively maintaining structural stability. These results demonstrate the superior delamination resistance provided by the covalently bonded heterogeneous interfacial layer.

### 2.7 Reliability Assessment of CPI‐PT10MMA Film

**FIGURE 7:**
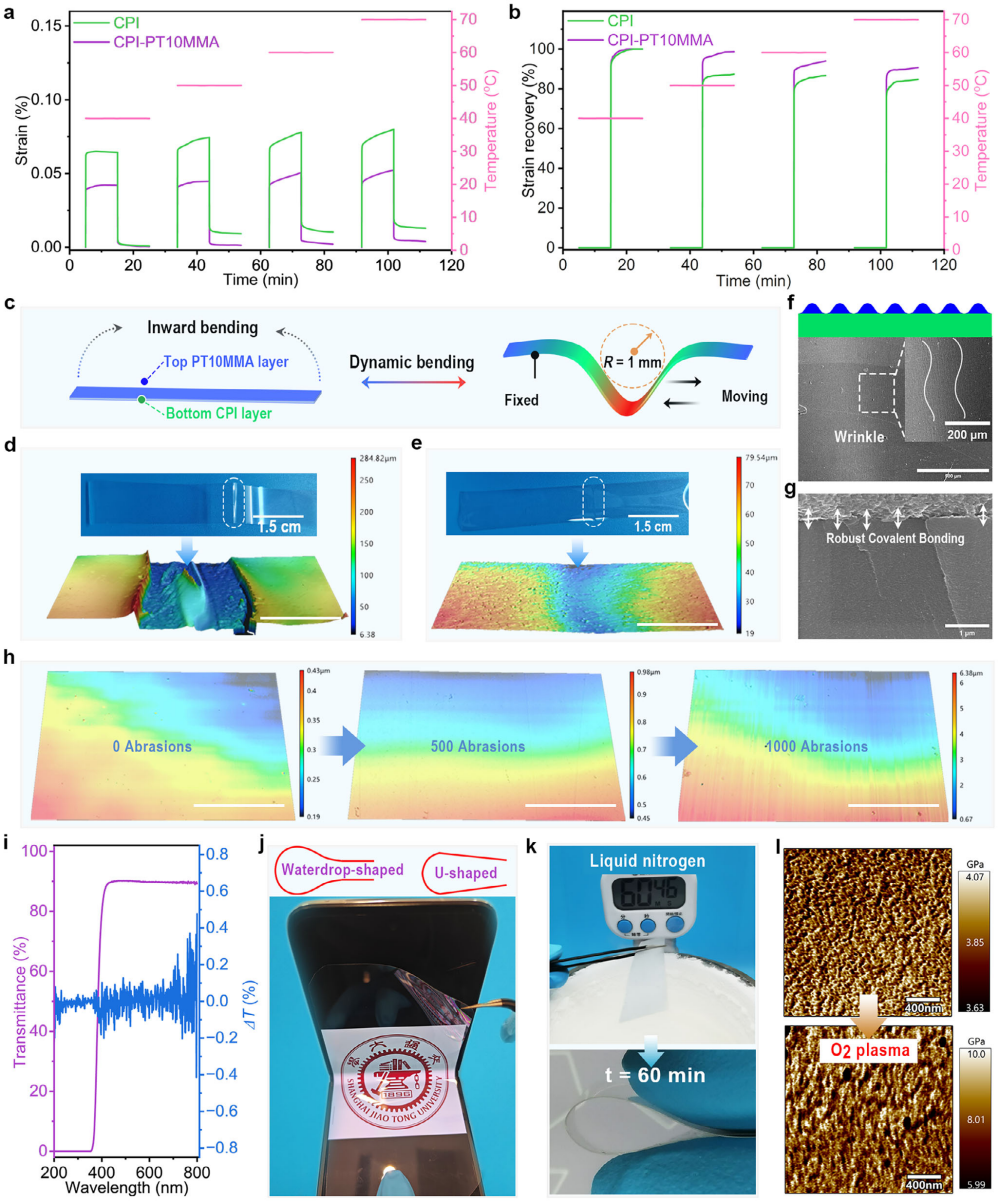
(a) Creep TTS curves of CPI and CPI‐PT10MMA film. (b) creep recovery curves of CPI and CPI‐PT10MMA film. (c) Schematic illustration of the dynamic folding for CPI‐PT10MMA film. The composite film is fixed at *R* = 1 mm, and the PT10MMA layer is on the inner side of the bend. (d, e) Photographs and Optical profiler 3D height images of (d) CPI film surfaces after 100 000 bending cycles and (e) CPI‐PT10MMA film after 200 000 bending cycles. The PT10MMA layer is on the inner side and the CPI layer on the outer side. Scale bar: 1 mm. (f, g) SEM images showing the (f) surface and (g) cross‐section of the CPI‐PT10MMA film. (h) 3D morphologies of CPI‐PT10MMA film after 0, 500, and 1000 abrasion cycles. Scale bar: 1 mm. (i) UV–vis spectra of CPI‐PT10MMA film after 1000 abrasion cycles. *ΔT* represents the difference in transmittance before and after abrasion, indicating the loss of transmittance. (j) Photograph of CPI‐PT10MMA film as FCW. (k) Low temperature resistance test of CPI‐PT10MMA film. (l) AFM modulus images of the PT10MMA layer before (top) and after (bottom) O_2_ plasma treatment.

To investigate the dimensional stability of the films, time‐temperature superposition (TTS) creep tests were conducted within the temperature range of 40°C–70°C. As shown in Figure 7a, the TTS curves for all films consist of two stages: the creep stage under a constant stress of 2 MPa and the recovery stage after stress removal. The CPI film displayed typical viscous flow behavior above 50°C, resulting in incomplete strain recovery upon removal of the external load and the consequent formation of permanent deformation. Compared to the CPI film, the CPI‐PT10MMA film exhibited lower creep strain in the temperature range of 40°C–70°C (Figure 7a; Figure S20). Below 50°C, the strain of the CPI‐PT10MMA film can achieve complete strain recovery, and even at 70°C, its strain recovery remained as high as 90.6%, surpassing the 84.5% observed for the CPI film (Figure 7b). This is because PT10MMA enhances the creep resistance of CPI through a robust heterogeneous interface. The dynamic folding performance of the films was subsequently evaluated using a homemade continuous folding apparatus. The films were fixed at *R* = 1 mm and subjected to continuous folding cycles (Figure 7c; Figure S21). The CPI film developed permanent creases after 100 000 bending cycles (Figure 7d; Figure S22a), whereas the CPI‐PT10MMA film only showed slight wrinkles on the PT10MMA layer after 200 000 bending cycles, with no structural failures observed (Figure 7e; Figure S22b). This behavior can be attributed to the continuous mechanical response of the covalently bonded heterointerface during bending, where the outer layer of the film reaches its tensile limit first, followed by the inner side attaining its compressive limit [13, 51]. As a result, the stress is first dissipated by the CPI layer, allowing the PT10MMA layer to develop only the initial morphology of buckling deformation (wrinkling) without progressing into catastrophic failure (Figure 7f). Furthermore, no localized damage or interfacial delamination was observed in the cross‐section of the CPI‐PT10MMA film after the bending test (Figure 7g), demonstrating its exceptional fatigue resistance. Despite the PT10MMA layer being only 30 µm, it still provides excellent pencil hardness (7H) and scratch resistance for the CPI‐PT10MMA film (Figure S23). After 500 abrasion cycles at a load of 2 N (contact area ∼2 cm^2^), the PT10MMA layer showed no detectable wear traces (Figure 7h; Figure S24). Even after 1000 abrasion cycles, the wear traces on the CPI‐PT10MMA surface are very shallow, and the transmittance shows negligible reduction after friction, thereby demonstrating its reliable capability for clear imaging (Figure 7i; Figures S24 and S25). In stark contrast, the CPI film exhibited severe wear after 5 abrasion cycles, significantly impairing display clarity and accurate imaging (Figures S25 and S26). Since the CPI‐PT10MMA film is a multifunctional integrated film, it holds promise for reducing the thickness and crease formation of display modules, as well as optimizing the weight and spatial volume of waterdrop‐ and U‐shaped hinges (Figure 7j). This is beneficial for the advancement of smart devices toward lighter, thinner, and more convenient designs.

The stability of CPI‐PT10MMA films under low‐temperature conditions and an oxygen plasma atmosphere was also evaluated separately. The CPI‐PT10MMA film exhibited no change in flexibility after being immersed in liquid nitrogen for 1 h and subsequently removed (Figure 7k; Figure S27). Following oxygen (O_2_) plasma treatment, the surface modulus of the PT10MMA layer surged from ∼4.01 to ∼9.6 GPa (Figure 7l). This enhancement is attributed to the removal of organic components within PT10MMA and the oxidation of the inorganic Si─O─Si core into a dense SiO_2_ passivation layer [66]. These results indicate that the CPI‐PT10MMA film holds promise for applications under extreme conditions.

## 3 Conclusion

In summary, we developed three functional POSS coatings (PT8MMA, PT10MMA, and PT12MMA) with identical chemical composition yet distinct structures through photo‐ or thermal crosslinking of their respective precursors. The optical transmittance of POSS coatings is negatively correlated with POSS cage sizes, while the hardness and hydrophobicity show opposite trends. Among these, PT10MMA coating achieved the best combination of optical, mechanical, and surface properties. Consequently, PT10MMA was covalently bonded to CPI to fabricate a bilayer‐structured film, CPI‐PT10MMA. The CPI‐PT10MMA film circumvents the inherent brittleness of inorganic materials while retaining their hardness and wear resistance, and mitigates the fatigue susceptibility of polymer films without compromising their flexibility. The robust heterogeneous interface stability facilitates synergistic interplay between the soft and hard layers, resulting in the emergence of novel functionalities that transcend the sum of the intrinsic performance capabilities of the individual components. As a result, in addition to its exceptional optical transparency, the CPI‐PT10MMA film achieves simultaneously excellent mechanical performance and dimensional stability, characterized by a hardness of 7H, wear resistance (>500 steel wool abrasion cycles), arbitrary bending and even folding capabilities, no creep under a constant stress of 2 MPa up to 50°C, and a strain recovery 90.6% at 70°C. And even after 200 000 bending cycles, this film with a heterogeneous interface maintains structural integrity without cracking, delamination, or permanent yielding. This work not only provides valuable insights into the selection of POSS structures with tailored functionalities but also offers a robust solution for achieving multifunctional integration in FCW films.

## 4 Experimental Section

### 4.1 Materials

4,4'‐(Hexafluoroisopropylidene) diphthalic anhydride (6FDA) and 2,2‐Bis (3‐amino‐4‐4 hydroxyphenyl) hexafluoropropane (6FAP) were obtained from ChinaTech Chemical Co., Ltd., China. 6FDA was dried in a vacuum oven for 24 h at 150°C prior to use. Tetrahydrofuran (THF, 99.9% purity), N‐Methyl‐2‐pyrrolidone (NMP), N, N‐dimethylacetamide (DMAc), and deuterated dimethyl sulfoxide (DMSO‐*d*_6_) with a trace amount of water (≤50 ppm) were acquired from J&K Scientific, China. Toluene (Tol, 98% purity), Acetone (Ace, ≥99.5% purity), and Concentrated hydrochloric acid (conc. HCl) were purchased from Sinopharm Chemical Reagent Co., Ltd., China. Toluene was refluxed over sodium and then distilled under nitrogen prior to use. 2,2‐Dimethoxy‐2‐phenylacetophenone (DMPA, 98.0% purity), Sodium methacrylate (98.0% purity), and 2‐Isocyanatoethyl Methacrylate (ICEMA, 98.0% purity) were purchased from Shanghai TCI Chemical Industry Co., Ltd., China. Absolute methanol (MeOH, ≥98.0% purity), Dichloromethane(DCM, water ≤50 ppm, 99.9% purity), Ethanol absolute (EtOH, water ≤50 ppm, 99.5% purity), Chloroform‐*d* (CDCl_3_, 98.0% purity), Ethyl acetate (EA, 99.5% purity), *n*‐Hexane (HEX, 97.0% purity), Magnesium sulfate (MgSO_4_, 98% purity), 2‐Propanol (IPA, water ≤50 ppm) and Xyxlene (water <50 ppm, 99.0% purity) were acquired from Shanghai Titan Chemical Reagent Co., Ltd., China. 3‐Chloropropyltrimethoxysilane (≥98.0% purity) and 1‐Methoxy‐2‐propyl acetate (PGMEA, ≥99.0% purity) were received from Shanghai Aladdin Biochemical Technology Co., Ltd., China. The glass (toughened and quartz glass) plates were thoroughly cleaned by ultra‐sonication in de‐ionized water, Ace, EtOH, and IPA, respectively, for about 10 min each. To ensure that the glass plates were free from surface contaminants, they were treated with oxygen plasma followed by rinsing in deionized water. Finally, the cleaned glass plate was dried with a nitrogen flow.

### 4.2 Synthesis of Materials

#### 4.2.1 Synthesis of Methacrylate‐Functionalized POSS (T8‐MMA, T10‐MMA, and T12‐MMA)

A three‐necked round‐bottom flask equipped with a reflux condenser and magnetic stir bar was charged with Cl‐POSS (11.0 g, 0.01 mol) and sodium methacrylate (12.3 g, 0.11 mol) under a nitrogen atmosphere. Anhydrous DMF (220 mL) was added, and the reaction mixture was stirred and heated to 70°C for 48 h. Upon completion, deionized water was introduced, and the resulting mixture was extracted with DCM. The organic layer was collected and dried over anhydrous Na_2_SO_4_. Purification via silica gel column chromatography using EA/HEX (3:7 v/v) as the eluent afforded three distinct fractions: T8‐MMA (1.5 g), T10‐MMA (3.2 g), and T12‐MMA (1.4 g). The synthesis details and characterization data of Cl‐POSS can be found in the Section S1, Figures S28 and S29.

#### 4.2.2 Synthesis of CPI

6FDA (23.1 g, 52.0 mmol) was added to a solution of 6FAP (18.3 g, 50.0 mmol) in DMAc (140.0 mL) at 0°C. The solution was then warmed to room temperature and magnetically stirred overnight under N_2_ atmosphere to form the viscose red polyimide acid (PAA). Xylene (62.0 mL) was added to the flask, and red or green PAA was thermally refluxed to form an imide ring at 180°C for 4 h. At the same time, the water generated from the ring‐closure reaction was separated as a xylene azeotrope. The resultant solution was cooled to room temperature and then added dropwise to a solution of methanol and water to obtain a precipitate. The precipitate was washed in distilled water three times, filtered, and dried in a vacuum oven at 60°C for 24 h to get PI, respectively.

ICEMA (7.5 g, 48.0 mmol) was added to a solution of PI (5.0 g) in DMAc (15.0 mL). The solution was then warmed to room temperature and magnetically stirred overnight under N_2_ atmosphere. The resultant solution was added dropwise to a solution of methanol and water to obtain a precipitate. The precipitate was washed in distilled water three times, filtered, and dried in a vacuum oven at 45°C for 24 h to get photo‐ and thermosensitive PI, abbreviated as CPI. For the characterization of CPI, please refer to Figure S30 and Table S5, respectively.

#### 4.2.3 Fabrication and Characterization of CPI‐PT10MMA Film

A CPI resin solution (DMAc solvent) with a solid content of 65.0% was initially cast onto a meticulously cleaned glass substrate. A sequential blade‐coating process was employed to prepare a uniform pre‐bottom layer wet film (200 µm), followed by sequential deposition of T10‐MMA solution (PGMEA solvent) with 15.0 wt.% onto the CPI layer using identical methodology to form the pre‐top layer. The resultant bilayer film precursor was transferred into a curing oven, which was first purged with nitrogen and then evacuated to a vacuum, followed by programmed sequential thermal treatments: 60°C for 30 min, 90°C for 20 min, 150°C for 30 min, 185°C for 30 min, with a heating rate of 5°C min^−1^. With CPI serving as a flexible substrate, T10‐MMA can achieve maximum curing without undergoing stress‐mediated brittle fracture. Upon completion of the curing program, the sample was gradually cooled to 40°C before being removed from the chamber. The CPI‐PT10MMA film was then carefully peeled off the glass substrate for further characterization. The obtained composite film comprises a CPI layer with a fixed thickness of 75 µm and a PT10MMA layer with an adjustable thickness.

## Author Contributions

H.Y.L. and Q.H.L. conceptualized and designed the project. H.Y.L., Z.H.L., and Y.H.W. carried out the experiments. H.Y.L. analyzed the data and wrote the first draft of the manuscript. J.N.Y. and S.F.X. assisted in data interpretation and revised the manuscript. Q.H.L. supervised the entire project, reviewed the manuscript, and managed resources and funding.

## Conflicts of Interest

The authors declare no conflicts of interest.

## Supporting information




**Supporting File**: advs74911‐sup‐0001‐SuppMat.docx.

## Data Availability

The data that support the findings of this study are available from the corresponding author upon reasonable request.
