# Supplementary material for: Molecularly Bridged Heterointerface Engineering: Designing Transparent, Wear‐Resistant, Hard yet Flexible Optoelectronic Protective Film
Source: Adv Sci (Weinh). 2026 Mar 19;13(31):e74911. doi: 10.1002/advs.74911 (PMC13588057; doi:10.1002/advs.74911)
Supplement: Supplementary file 1 [file ADVS-13-e74911-s001.docx]

Supporting Information for

**Molecularly Bridged Heterointerface Engineering: Designing Transparent, Wear-Resistant, Hard yet Flexible Optoelectronic Protective Film**

Huiyang Lu^1^, Zihui Liu^2^, Jianan Yuan^1^, Yunhao Wei^1^, Shuangfei Xiang^3*^, and Qinghua Lu^1,4*^

*^1^State Key Laboratory of Synergistic Chem-Bio Synthesis, School of Chemistry and Chemical Engineering, Shanghai Jiao Tong University, Shanghai 200240, China*

*^2^School of Chemical Science and Engineering, Tongji University, Shanghai 200092, China*

*^3^School of Materials Science and Engineering, Zhejiang Sci-Tech University, Hangzhou 310018, China*

*^4^State Key Laboratory of Micro-Nano Engineering Science, Shanghai Jiao Tong University, 800 Dongchuan Road, Shanghai 200240, China*

^*^Corresponding author: [xiang@zstu.edu.cn](mailto:xiang@zstu.edu.cn) (Shuangfei Xiang); [qhlu@sjtu.edu.cn](mailto:qhlu@sjtu.edu.cn) (Qinghua Lu)

**This supplementary information includes:
Experimental Section**

Scheme S1

Figures S1 to S30

Tables S1 to S5

References

**Experimental Section**

**Synthesis of Materials**

**Scheme S1. Synthesis of T8-MMA, T10-MMA, and T12-MMA**

***Synthesis of octa(3-chloropropyl)octasilsesquioxane (Cl-POSS)***^[1, 2]^**:** A dual-necked round-bottom flask equipped with a dropping funnel and magnetic stir bar was charged with anhydrous methanol (357.0 g, 450.0 mL) and concentrated hydrochloric acid (17.8 g, 0.18 mol). Subsequently, (3-chloropropyl)trimethoxysilane (45.0 g, 0.23 mmol) was added dropwise through the addition funnel over 30 minutes under vigorous stirring while maintaining the reaction temperature at 40°C. After continuous stirring for two weeks, a large white precipitate was obtained. The resultant solid was isolated by vacuum filtration and sequentially washed with deionized water followed by methanol. The purified product was subsequently dried under vacuum at 30°C to afford Cl-POSS as a white solid (11.04 g, 34% yield). ^1^H NMR (400 MHz, CDCl_3_; ppm) δ 3.53 (t, *J* = 6.6 Hz, 16H, SiCH_2_), 1.87 (m, *J* = 6.9 Hz, 16H, CH_2_), 0.80 (dd, *J* = 9.8, 6.6 Hz, 16H, CH_2_Cl). ^29^Si NMR (79 MHz, CDCl_3_; ppm) δ -67.10.

***Preparation of PT8MMA Coating:*** A 50 µm-thick heat-resistant polyimide tape was used to create a “square” frame-shaped recessed mold on a clean quartz substrate (2 × 2 cm^2^). Subsequently, 0.1 mL of T8-MMA (PGEMA solvent) solution (1 g mL^-1^) was poured into the recess. The sample was then placed in a vacuum oven at 60 °C to remove the solvent. This process of adding the T8-MMA solution and solvent evaporation was repeated until the sample thickness exceeded the recess depth by approximately 1 mm. The quartz substrate with the sample was then placed on a hot press stage and thermally cured under 0.2 MPa pressure, following a stepwise heating protocol: 70 °C for 30 min, 110 °C for 30 min, 150 °C for 30 min, and 180 °C for 60 min. After the thermal treatment, the sample was slowly cooled to room temperature, yielding a ~50 µm-thick coating.

***Preparation of PT10MMA and PT12MMA Coatings:*** A homogeneous mixture was prepared by dissolving 100.0 mg of T10-MMA or T12-MMA and 1.0 mg (1 wt.%) photoinitiator DMPA in 0.35 mL of PGEMA under stirring. The solution was filtered through a 0.22 µm organic membrane filter and then degassed in a vacuum oven at 25 °C for 8 h. Under a nitrogen atmosphere at 25 °C, 0.2 mL of the solution was cast onto a clean quartz substrate (3 × 3 cm^2^). After solvent evaporation (confirmed by mass stabilization within 30 min for the T10-MMA-coated quartz substrate), the sample was cured under a 50 W UV lamp for 6 min, resulting in a ~50 µm-thick coating.

**Characterization**

Q800 instrument (TA, USA) was utilized for performing the dynamic mechanical analysis (DMA) and the creep behavior of the films. The creep stress was set at 2.0 MPa, which is much lower than the yield strength of the samples. Wide angle X-ray diffraction (WAXD) tests for all samples were performed on a Rigaku Miniflex 600 instrument (Rigaku, Japan) with Cu-Kα radiation (λ = 1.5406 Å), and 2θ scan data were measured and collected in the 5° to 50°range. The thermodynamic properties of all samples were characterized under a nitrogen atmosphere using a heating and cooling rate of 10 °C min^−1^ with a Discovery DSC 800 (TA, USA). Gel Permeation Chromatography (GPC) LC-20AD instrument (Shimadzu, Japan) was employed for determining the relative molecular weight of polyimides, polystyrene was used as the standard and N, N-Dimethylformamide (DMF) was used as the eluent. The metallographic microscope (MX4RT, HuiGuang Technology Co., Ltd, China), and Ultra-Depth 3D Microscope VHX-7000 (KEYENCE, Japan) were used to observe the optical images of coatings and films. Polarized Optical Microscopy (POM) images were obtained by Leica DM LP (Leica, Germany). The mechanical properties of the films were tested using a CMT1104 (SUST, China). Nanoindentation tests on the coatings were conducted using an Agilent G200 The dynamic bending test was performed on a homemade continuous bending apparatus. The minimum bending *R* was estimated by constantly decreasing the gap of U-shape before cracks appeared. The scratch resistance test was carried out on a scratch resistance tester HSR-2M (Changzhou Ruipin Precision Instrument Co., Ltd., China). The end of the scratching rod was wrapped with steel wool (grade #0000), and the sample was scratched back and forth under a pressure of ~10.0 kPa. All pencil hardness tests were evaluated using an Elecometer 501 Pencil Hardness Tester (Elcometer, U.K.) based on the ASTM D3363 test method. The fracture surface and surface morphology of the sample were analyzed by Scanning Electron Microscope (SEM) (Apreo 2S, Thermo Scientific, USA; TESCAN, Czech Republic), and the distribution of elements on the surface of the sample was characterized by energy dispersive X-ray spectroscopy probes in RISE-MAGN (TESCAN, Czech Republic). All samples were sputter-coated with a thin layer of gold before observation. UV-1800 spectrophotometer (Shimadzu, Japan) was utilized to evaluate the optical properties of all films and coatings. The contact angle (WCA and DCA) and surface energy (γ_s_) were measured using a DSA100 contact angle meter produced by KRUSS, Germany. The volume of each droplet was 5 μL (H_2_O and CH_2_I_2_) at room temperature, and the surface energy of all samples was calculated using the Owens-Wendt-Rabel-Kaelble (OWRK) method^[3, 4]^. Atomic force microscope (AFM) nanomechanical measurement was conducted on Oxford MFP-3D (Oxford Instruments, UK) to characterize the topography and mechanical properties of samples. Nuclear magnetic resonance (NMR) data were recorded on a Bruker 400 (Bruker, AVANCE III HD 400) spectrometer at 298 K. Transmission electron microscopy (TEM) JEM-2100F (JEOL, Japan) was employed to evaluate the internal structure of all samples. In situ synchrotron radiation small angle X-ray scattering (SAXS) measurements were performed at the beamline BL16B1 at Shanghai Synchrotron Radiation Facility (SSRF, China). In situ Fourier Transform Infrared Spectroscopy was systematically employed to monitor POSS Coating Curing with a METTLER TOLEDO ReactIR 15 system and prepare CPI-PT10MMA film with a Thermo Scientific Nicolet iS50 instrument, respectively. The degree of curing was quantitatively determined by monitoring the relative intensity changes of characteristic absorption bands, specifically the C=O and the C=C, as previously reported.^[5]^ A Romulus IV (Quad Group Inc., Japan) stud pull tester was used to test the bonding strength between coatings and films.

**Figure S1.** ^13^C NMR analysis of T8-MMA, T10-MMA, and T12-MMA

**Figure S2.** XRD pattern of T8-MMA.

**Figure S3.** Methacrylate conversion of T10-MMA and T12-MMA obtained by thermal curing as a function of curing temperature.

**Figure S4.** The photograph of PT8MMA coatings cured at different temperatures from T8-MMA. The PT8MMA coating maintains structural integrity without fracturing exclusively when processed at temperatures below 185 °C (30 min) accompanied by methacrylate conversion rates remaining under 78.8%, as determined by FTIR spectroscopic monitoring.

**Figure S5.** The WAXD pattern of PT8MMA reveals the presence of two broad amorphous peaks, as opposed to the crystalline peaks observed in T8-MMA. These peaks are attributed to the POSS cage and its substituents, respectively, indicating that the coating is a hybrid material at the nanoscale length.

**Figure S6.** PT10MMA as cover window coating. The PT10MMA coating demonstrates exceptionally high optical transparency, ensuring no interference with display visibility, while simultaneously providing a tactile sensation akin to that of glass.

**Figure S7.** a,b) Cross-sectional structure of PT12MMA coatings obtained with PGMEA as solvent (a) 100um thick and (b) 20um thick. c,d) Surface structure of PT12MMA coatings obtained with DCM as solvent (c) 20um thick and (d) 100um thick.

**Figure S8.** UV–vis spectra of PT12MMA. Further reduction in the film thickness (35 µm) and optimization of the solvent can marginally enhance the transmittance; however, the film still exhibited severe haze.

**Figure S9.** Water (WCA) and oil (DCA) contact angle of PT8MMA, PT10MMA, and PT12MMA coatings.

**Figure S10.** Photographs of the PT10MMA coating at various stages of abrasion using steel wool under a pressure of ⁓10.0 kPa for 1,000 cycles. Scale bar: 1.5 cm.

**Figure S11.** a) Pencil hardness tester. b) Pencil hardness of PT8MMA, PT10MMA, and PT12MMA coatings as a function of curing time.

**Figure S12.** PT10MMA serves as a coating for the CPI film. Due to weak adhesion, delamination occurs between the CPI and PT10MMA layers.

**Figure S13.** Reflectance of PT10MMA, CPI-PT10MMA and CPI coatings.

**Figure S14.** a) The crystallization of the films was examined using polarized light microscopy. (b) OM and (c) POM images of CPI film. (d) OM and (e) POM images of CPI-PT10MMA film. Both the CPI film and CPI-PT10MMA (the PT10MMA layer oriented upward) film exhibited no detectable crystallinity, as evidenced by the absence of birefringence patterns (light and dark variations or color patterns) under cross-polarized illumination. This observation aligns with the amorphous phase characteristics confirmed by prior TEM analyses. Scale bar: 200 µm.

**Figure S15.** a) HAADF-STEM image of CPI-PT10MMA film. b,c) EDX elemental mappings of (b) O and (c) F elements in CPI-PT10MMA film.

**Figure S16.** Thickness measurement for samples used in pull-off test. The thickness of the PT10MMA layer is determined by the difference between the thickness of the CPI-PT10MMA film and the thickness of the CPI film.

**Figure S17.** Adhesion strength testing of CPI-PT10MMA films with varying PT10MMA layer thicknesses. a) ~250 µm. b) ~150 µm. c) ~100 µm. c) ~50 µm. Despite the strong interfacial bonding strength between PT10MMA and CPI, the excessive thickness of the PT10MMA layer (>50 μm) induces brittle fracture under external force, thereby compromising the flexibility and mechanical durability of the CPI-PT10MMA composite film.

**Figure S18.** Adhesion strength (interfacial bonding strength) between the PT10MMA layer and CPI for varying PT10MMA layer thicknesses.

**Figure S19.** Comparison of heterogeneous interfacial adhesion strength between covalently bonded CPI-PT10MMA films and representative non-bonded or hydrogen-bonded polysiloxane coatings on polymer substrates. (PET and CPI).^[5-11]^

**Figure S20.** The maximum creep strain of both CPI and CPI-PT10MMA films in the range of 40–70 °C.

**Figure S21.** Dynamic bending test of CPI-PT10MMA film at *R* = 1mm.

**Figure S22.** a,b) OM images of (a) CPI film surfaces after 100,000 bending cycles and (b) CPI-PT10MMA film after 200,000 bending cycles.

 **Figure S23.** Pencil hardness of CPI (75 µm), CPI-PT10MMA (115 µm) and PT10MMA (30 µm) coatings.

**Figure S24.** OM images of CPI-PT10MMA film after (a) 500 abrasion cycles, (b) 2 abrasion cycles, and (c) 5 abrasion cycles. Size: 2900 × 2200 µm.

**Figure S25.** The friction-treated films were applied onto the display screen. a) CPI film after 5 abrasion cycles and b) CPI-PT10MMA film after 1000 abrasion cycles. Scale bar: 1.5 cm.

**Figure S26.** a-c) 3D morphology and d-e) OM images of CPI film after (a,d) 0 abrasion cycles, (b,e) 2 abrasion cycles, and (c,f) 5 abrasion cycles. Size: 2900 × 2200 µm.

**Figure S27.** Low temperature resistance test of CPI-PT10MMA film. CPI-PT10MMA film can recover to its original state and achieve large deformation after being immersed in liquid nitrogen for 1 hour.

**Figure S28.** ^1^H NMR spectra of Cl-POSS.

**Figure S29.** ^13^C NMR spectra of Cl-POSS

**Figure S30.** ^1^H NMR spectra of CPI. ICEMA grafting rate was calculated from the ratio of peak (a) (-OH) to peak (k) (=CH_2_).

**Table S1.** The optimization of solvents for preparing PT12MMA coating.

| **Solvents** | **NMP** | **DCM** | **Tol** | **THF** | **EtOH** |
| --- | --- | --- | --- | --- | --- |
| **Mixture solution^a)^** | ++ | ++ | ++ | ++ | ++ |
| **Solvent evaporated** | ++ | – | ++ | – | + |
| ***T*_500nm_ (%)^b^** | 72.0 | 56.2 | 62.4 | 54.7 | 58.3 |

a) The mixed solution includes T12-MMA, DMPA, and a solvent to be optimized. b: The transmittance of the UV-cured PT12MMA coating at 550 nm wavelength was averaged over three measurements. All PT12MMA coating thickness measurements fell within the range of 92 to 100 µm; “++”: The formulation exhibited a colorless, transparent, and optically homogeneous appearance; +: The formulation exhibited localized turbidity; “–”: The formulation exhibited localized turbidity, accompanied by slight haze formation.

**Table S2.** Mechanical properties determined from the nanoindentation tests

| **Samples** | | ***H***  **(GPa)** | ***E^*^***  **(GPa)** | ***W*_e_**  **(%)** | ***H*/*E^*^***  **(%)** | ***H^3^*/*E^*2^* (MPa)** |
| --- | --- | --- | --- | --- | --- | --- |
| **This work^a)^** | **PT8MMA** | 0.38 | 4.1 | 85.20 | 9.27 | 3.26 |
|  | **PT10MMA** | 0.58 | 5.2 | 86.13 | 11.15 | 7.22 |
|  | **PT12MMA** | 0.61 | 5.8 | 75.32 | 10.52 | 6.75 |
| Others: The data for the remaining specimens, encompassing polymers, metals, elastomers, and ceramics, were derived from *Ref*.^[12]^ | | | | | | |

a) Data are the average of three measurements.

**Table S3.** Mechanical properties of CPI

| Sample | Young's modulus, *E*  (GPa) | Elongation at break  (%) |
| --- | --- | --- |
| CPI | 3.8 | 5.7 |

**Table S4.** EDX elemental data of element contents of CPI-PT10MMA

| Element | Family | Atomic Fraction (%) | Atomic Error (%) | Mass Fraction (%) | Mass Error  (%) | Fit Error  (%) |
| --- | --- | --- | --- | --- | --- | --- |
| C | K | 46.28 | 3.67 | 38.21 | 3.41 | 1.36 |
| N | K | 3.24 | 0.72 | 3.12 | 0.71 | 7.91 |
| O | K | 37.03 | 4.78 | 40.74 | 5.05 | 0.95 |
| F | K | 12.88 | 2.48 | 16.82 | 3.17 | 4.08 |
| Si | K | 0.57 | 0.15 | 1.11 | 0.3 | 17.06 |

**Table S5.** GPC data and ICEMA grafting ratio of CPI.

| **Samples^a)^** | ***M*_w_**  **(× 10^4^ g mol^-1^)** | ***M_n_***  **(× 10^4^ g mol^-1^)** | **ICEMA grafting ratio (%)** |
| --- | --- | --- | --- |
| **CPI-1** | 0.91 | 0.55 | 80.1 |
| **CPI-2** | 3.10 | 1.82 | 54.6 |
| **CPI-3** | 6.30 | 3.65 | 31.2 |

a) High-molecular-weight CPI can provide better mechanical and thermal properties, while a lower ICEMA grafting ratio contributes to a reduced yellowness index. Therefore, the CPI-3 (denoted as CPI) was selected to construct a bilayer composite film with T10MMA.

**Reference**

[1]	B. Marciniec, M. Dutkiewicz, H. Maciejewski, M. Kubicki, *Organometallics* **2008**, 27, 793.

[2]	U. Dittmar, B. J. Hendan, U. Flörke, H. C. Marsmann, *J. Organomet. Chem.* **1995**, 489, 185.

[3]	D. H. Kaelble, *J. Adhes.* **1970**, 2, 66.

[4]	D. K. Owens, R. C. Wendt, *J. Appl. Polym. Sci.* **1969**, 13, 1741.

[5]	X. Lin, H.-T. Li, M.-X. Nie, S.-R. Fu, Y. Li, Q. Zhang, F. Chen, D. Han, Q. Fu, *ACS Appl. Mater. Interfaces* **2022**, 14, 39432.

[6]	K. Pan, C. Liu, Z. Zhu, T. Feng, S. Tao, B. Yang, *ACS Appl. Mater. Interfaces* **2022**, 14, 14504.

[7]	Y. Zhang, Z. Chen, H. Zheng, R. Chen, C. Ma, G. Zhang, *Adv. Sci.* **2022**, 9, 2200268.

[8]	R. Chen, Y. Zhang, Q. Xie, Z. Chen, C. Ma, G. Zhang, *Adv. Funct. Mater.* **2021**, 31, 2011145.

[9]	X. Lin, C. Y. Li, L. X. Liang, Q. Y. Guo, Y. Zhang, S. R. Fu, Q. Zhang, F. Chen, D. Han, Q. Fu, *Nat. Commun.* **2024**, 15, 9680.

[10]	P. Zhang, G. Zhang, J. Pan, C. Ma, G. Zhang, *ACS Appl. Mater. Interfaces* **2023**, 15, 5998.

[11]	J. Zhu, Y. Ma, J. Wei, Y. Hu, H. Gou, H. Wang, W. Fang, H. Fan, J. Wan, *Prog. Org. Coat.* **2025**, 201, 109101.

[12]	W. Fang, Z. Mu, Y. He, K. Kong, K. Jiang, R. Tang, Z. Liu, *Nature* **2023**, 619, 293.
